# *ERCC1* polymorphisms as prognostic markers in T4 breast cancer patients treated with platinum-based chemotherapy

**DOI:** 10.1186/s12967-014-0272-4

**Published:** 2014-09-25

**Authors:** Grazia Palomba, Francesco Atzori, Mario Budroni, MariaNeve Ombra, Antonio Cossu, MariaCristina Sini, Valeria Pusceddu, Bruno Massidda, Barbara Frau, Francesca Notari, MariaTeresa Ionta, Giuseppe Palmieri

**Affiliations:** Institute of Biomolecular Chemistry (ICB), National Research Council (CNR), Traversa La Crucca, 3 - Località Baldinca Li Punti, 07100 Sassari, (SS) Italy; Department of Medical Oncology, Hospital-University of Cagliari, Cagliari, Italy; Epidemiology Unit, Azienda Sanitaria Locale 1, Sassari, Italy; Institute of Food Science (ISA), National Research Council (CNR), Avellino, Italy; Institute of Pathology, Azienda Ospedaliero Universitaria, Sassari, Italy

**Keywords:** T4 breast cancer, Genetic polymorphism, Platinum-based chemoterapy, Prognosis

## Abstract

**Background:**

Polymorphisms in the *excision repair cross-complimentary group 1* (*ERCC1*) gene have been involved in the prognosis of various cancers. In the present study, we evaluated the prognostic role of the two most common *ERCC1* polymorphisms in patients with T4 breast cancer receiving platinum-based chemotherapy.

**Methods:**

A total of 47 patients with T4 breast cancer undergoing treatment with a platinum-based regimen were collected and followed up (median 159 months; range, 42–239 months). *ERCC1* C8092A (rs3212986) and T19007C (rs11615) polymorphisms were genotyped, using an automated sequencing approach. The same series was screened for *BRCA1/2* mutations by DHPLC analysis and DNA sequencing.

**Results:**

Among the tested patients, 16 (34%) and 25 (53%) presented the 8092A (homo-zygosity A/A or heterozygosity A/C) and the 19007C (homozygosity C/C or heterozygosity C/T) genotypes, respectively. The 8092A and 19007C genotypes in *ERCC1* were significantly associated with overall survival in T4 breast cancer patients treated with chemotherapy containing platinum (p-values = 0.036 and 0.004, respectively). Univariate and multivariate Cox regression analyses showed that combination of 8092A and 19007C genotypes acts as a significant prognostic factor in women with T4 breast cancer receiving platinum-based chemotherapy (p-values = 0.022 and 0.049, respectively). Two (4.3%) out of 47 cases were found to carry *BRCA1/2* mutations; they presented the highest overall survival rates into the series.

**Conclusions:**

The *ERCC1* 8092A and 19007C genotypes or their combination may predict a favorable prognosis in T4 breast cancer patients undergoing a platinum-based treatment. Further large-scale, prospective studies are needed to validate our findings.

## Introduction

Breast cancer remains the most frequent tumor and the leading cause of cancer-related death among the female population worldwide [1].

Locally advanced breast cancer (LABC) represents a heterogeneous group of diseases associated with a poor prognosis. According to the International Union Against Cancer (UICC)/American Joint Committee on Cancer (AJCC) TNM staging system, primary breast cancers with extension to the skin, with or without lymph node involvement, and without distant metastases (T4 N0-2 M0), may be included in stage III and considered as LABC [2].

Overall, patients with LABC - including cases presenting an inflammatory disease and, mostly, those carrying a triple-negative breast cancer - are particularly responsive to DNA-damaging agents such as platinum compounds; for this reason, platinum-based chemotherapy is frequently used as neoadjuvant treatment in such disease types [3-6]. The cytotoxic effect of platinum drugs is ascribed to the formation of bulky platinum-DNA adducts, which block replication and transcription through inter-strand cross-link of the two DNA strands, leading to cancer cell death. These adducts are recognized and removed, with subsequent repair of the inter-strand cross-links in DNA, by factors of the nucleotide excision repair (NER) pathway [7]. Recent published data have revealed that single nucleotide polymorphisms (SNPs) in DNA repair genes may represent an underlying molecular mechanism that explains inter-individual variation in DNA repair capacity [7].

The excision repair cross-complementing 1 (ERCC1) is one of the key effector of the NER pathway. This enzyme acts as a DNA damage repair gene, which is essential for the removal of platinum-DNA adducts as well as for recognition and correction of DNA damage [8]. Functional variants in genes involved in the DNA repair pathway may be important determinants of platinum response [9]. Therefore, ERCC1 mRNA and protein expression level or *ERCC1* gene polymorphisms may be used to predict the outcome in patients receiving platinum-based chemotherapy [10,11].

C8092A (rs3212986) and T19007C (rs11615) are two common polymorphisms in *ERCC1* gene. The C8092A polymorphism is located in the 3′untranslated region of the gene and may affect ERCC1 messenger RNA stability. The synonymous T19007C polymorphism at codon 118 (Asn118Asn) converting a common codon usage (AAC) to an infrequent one (AAT), both coding for asparagine, has been proposed to impair ERCC1 translation and to affect the response to chemotherapy [12].

In recent years, many studies focused on the association between clinical behavior of different types of cancer and specific SNPs in genes involved in DNA repair, including genes of the NER pathway. Although controversial results have been reported for association between polymorphisms of ERCC1 and cancer outcome (see below), increasing and more consistent evidence suggest a relationship between the level of ERCC1 expression and the response to chemotherapy. Higher mRNA levels of ERCC1 are associated with lack of platinum response in advanced lung [13,14], ovarian [15], bladder [16], and gastrointestinal [17-19] cancers. Consequently, lower ERCC1 mRNA levels have been found consistently associated with an improved tumor response using platinum-containing compounds [8,14,17].

Polymorphisms in *ERCC1* gene have been studied extensively, with controversial results. A study indicates that the codon 118 C > T polymorphism was not associated with clinical outcome in women with stage III ovarian cancer, whereas the C8092A polymorphism was an independent predictor of progression-free survival and overall survival (OS) in the same series of patients [20]. In contrast to this findings, additional reports indicated that the C/C genotype at codon 118 of ERCC1 expression may predict the response to platinum in either the same type of ovarian cancer [15] or other malignancies [12,21]. Overall, the T19007C polymorphism showed a controversial association with clinical outcome (in terms of either tumor response or OS) among different types of cancers [22-24]. The C8092A polymorphism was instead associated with a more favorable outcome in head and neck squamous cell carcinoma and advanced non small cell lung cancer patients [25,26]. Finally, the accumulated evidence provided by a meta-analysis of the literature clearly indicated that ERCC1 T19007C and C8092A polymorphisms might not act as risk factors for cancer [27].

There are differences in survival among patients who begin treatment in a similar disease status and genetic factors may influence the effectiveness of therapy. For this reason the availability of new biomarkers that can accurately predict the prognosis and patient response to the treatment is a central issue to improve therapeutic strategies.

The aim of the present study was to investigate whether the *ERCC1* 19007C > T and 8092C > A polymorphisms may influence the clinical outcome in response to treatment with platinum within a well-characterized cohort of patients with T4 breast carcinoma and long follow-up evaluation. Since germline mutations in *BRCA1* and, to a less extent, *BRCA2* genes have been found in a variable proportion (ranging from 10% to 30%) of patients with LABC or, mostly, triple-negative breast cancer [28], and such gene dysfunctions seem to be associated with prognosis [29], we also evaluated the prevalence of *BRCA1-2* mutations in our series.

## Materials and methods

### Samples

Germline DNA samples of 47 consecutive patients with T4 breast cancer [12 (26%) of them classified as inflammatory breast cancer (T4d-IBC)] were included into the study. Cases were enrolled between 1995 and 2004, and observed up to July 2013 for an overall median of 161 months (range, 13–242 months). Patients were assessed by physical examination and mammography, confirmed via core-needle biopsy. All patients completed a treatment plan including neoadjuvant platinum-based chemotherapy, surgery, radiation therapy, adjuvant chemotherapy, and hormone therapy, when indicated (see below). All patients were of Sardinian origin; the median age at diagnosis was 51 years (range 33–67 years). Patients’ characteristics are summarized in Table 1.

**Table 1:** **Patients’ characteristics and clinical features**

***Characteristics***	***Patients (N = 47)***	***%***
**Age** (year)		
*Median 51 (range 33–67)*		
<50	21	*45*
>50	26	*55*
**Histologic type**		
Ductal carcinoma	29	*62*
Lobular carcinoma	10	*21*
Other	1	*2*
Unknown	7	*15*
**Tumor grading**		
G2	33	*70*
G3	14	*30*
**Tumor extent**		
T4a,b,c	35	*74*
T4d	12	*26*
**Lymph node involvement**		
N0	6	*13*
N+	41	*87*
**ER/PgR status**		
ER positive/ER negative	23/24	*49/51*
PR positive/PR negative	16/31	*34/66*
**HER2 status**		
HER-2 positive	13	*28*
HER-2 negative	34	*72*
**Triple-negative status**		
Present	19	*40*
Absent	28	*60*
**Proliferative index**		
Ki67 positive	24	*51*
Ki67 negative	23	*49*

The study was approved by the Review Board at the University of Cagliari (Prot. 102/1996). A written informed consent was obtained for using tissue specimens in molecular analyses.

### Treatment plan

All 47 patients were treated with primary chemotherapy using PEV [cisplatin, epirubicin, vinorelbine; N = 42 (89%)] or PE-TXT [cisplatin, epirubicin, docetaxel; N = 5 (11%)]. After completing the neoadjuvant chemotherapy, patients underwent breast-conserving surgery (17/47; 36%) or radical mastectomy (30/47; 64%). Postoperative adjuvant chemotherapy consisted of six cycles of CMF (cyclophosphamide, methotrexate, fluorouracil). Locoregional radiotherapy was performed during the fourth course of CMF. After completing adjuvant chemotherapy, patients with hormone receptor-positive tumours received tamoxifen for 5 years.

Clinical evaluations were performed every 3 months for 2 years and every 6 months thereafter. Instrumental examinations (e.g., mammography, liver ultrasound, chest X-ray, bone scan, and echocardiogram) were performed every 6 months for the first 2 years, and every 12 months thereafter.

### Genetic analysis

Genomic DNA from all patients was isolated from peripheral blood nucleated cells, using standard methods, and then screened for the C > A and C > T polymorphisms at positions 8092 and 19007, respectively, of the *ERCC1* gene, using an automated direct sequencing approach. Primers for polymerase chain reaction (PCR) assays and protocols for PCR-based amplification have been previously described [30].

The entire coding sequences and intron-exon boundaries of the *BRCA1* and *BRCA2* genes were screened for germline mutations among all patients from our series. Mutation analysis was performed by denaturing high-performance liquid chromatography (DHPLC), followed by automated sequencing, as we previously reported [31,32]. Briefly, DHPLC analysis was carried out with the Wave® nucleic acid fragment analysis system (Transgenomic, Santa Clara, CA). Suspected variants are visualized as a characteristic pattern of peaks corresponding to the mixture of homo- and heteroduplex formed when wild-type and mutant DNA are hybridized. Abnormal PCR products identified by DHPLC analysis were directly sequenced using an automated fluorescence-cycle sequencer (ABIPRISM 3130, Life Technologies/ThermoFisher Scientific, Waltham, MA, USA).

### Statistical analysis

Odds ratios of carrying the *ERCC1* 19007C > T and 8092C > A polymorphisms were estimated by the logistic regression model and reported with 95% confidence interval (95% CI). Analyses were performed with the statistical package SPSS/7.5 for Windows.

## Results

### Patient characteristics and treatments

Forty-seven patients with diagnosis of T4 breast carcinoma (T4-N0/2-M0, according to the TNM classification by Sobin *et al.* [33]) were included into the study. Considering the disease staging system [33], all cases from our series were classified with the highest stage of non-metastatic disease (Stage IIIB). Among them, 19 (40%) patients presented the subtype of triple-negative breast cancer, with the characteristics of estrogen receptor (ER) negative, progesterone receptor (PR) negative, and human epidermal growth factor receptor-2 (HER-2) negative (Table 1). All the patients received platinum-based chemotherapy as the neoadjuvant treatment: nearly all of them (89%; see Methods) received PEV treatment. After surgery, adjuvant treatment included CMF chemotherapy for six cycles and locoregional radiotherapy (see Methods).

### C9082A and T19007C polymorphisms in ERCC1

Genomic DNA samples were extracted from peripheral blood mononuclear cells and single nucleotide polymorphisms, C8092A (rs3212986) and T19007C (rs11615), in the *ERCC1* gene were analyzed using an automated sequencing approach.

For C8092A, 16 (34%) patients presented the A genotype (3 AA, 13 AC), whereas 31 (66%) cases presented the C genotype (CC). For T19007C, the C (12 CC, 13 CT) and the T (TT) genotypes were found in 25 (53%) and 22 (47%) patients, respectively. Both distributions were in Hardy-Weinberg equilibrium. Considering the two polymorphisms the distribution of AC, AT, CC and CT genotypes was 25.5%, 8.5%, 28%, and 38%, respectively.

### BRCA mutation analysis

The germline DNA samples from the T4 breast carcinoma patients of the present study was analyzed for mutations in both the *BRCA1* and *BRCA2* genes, as previously described [31,32]. Among the 47 cases of the series, two germline coding region mutations of known functional significance in either *BRCA1* or *BRCA2* were detected in two (4.3%) patients. Both mutations, BRCA1 (2CA) 916delTT and BRCA2 3951del3insAT, were absent in normal genomic DNA from 103 unrelated healthy individuals (corresponding to 206 control chromosomes) and were classified as disease-causing variants due to their predicted effects on proteins.

### Survival analysis

As of July 2013, 18 (38%) patients have died due to disease, with the median overall survival of the whole sample being 108 months and the median follow-up of live patients of 153 months.

According to the C8092A polymorphism status, the median OS was significantly higher for patients carrying the A (AA + AC) genotype as compared to those with the C (CC) genotype (123.5 vs.101.6 months; p = 0.036). Analogously, a significantly strong association between the T19007C polymorphism and overall survival was observed; the median OS was 131 months for carriers of the C (CC + CT) genotypes and 66.5 months for TT homozygotes (p = 0.004) (Table 2A). When the combination of both *ERCC1* polymorphisms were included in univariate analysis, carriers of the combined 8092A/19007C genotype presented an overall survival significantly longer than that of patients carrying the combined C8092/T19007 genotype (143.5 vs. 91.7 months; p-value = 0.022) (Table 2B). No association between T19007 or C8092A *ERCC1* polymorphisms and age at diagnosis, tumor grade, histology, menopausal condition was detected (not shown).

**Table 2:** **Logistic regression analysis for overall survival and ERCC1 polymorphisms**

**A**				
	**Odds ratio**	**95% CI lower**	**95% CI upper**	**p-value**
**C8092A**	**1.957**	**1.276**	**5.675**	**0.036**
**T19007C**	**3.875**	**1.865**	**18.851**	**0.004**
**B**				
	**Odds ratio**	**95% CI lower**	**95% CI upper**	**p-value**
C8092 + T19007	2.479	0.538	11.421	0.244
**8092A + 19007C**	**5.609**	**1.278**	**24.622**	**0.022**

Single (A) or combined (B) ERCC1 genotypes have been considered for statistical analysis. Significant associations are in bold. CI, confidence interval.

**Figure 1:**
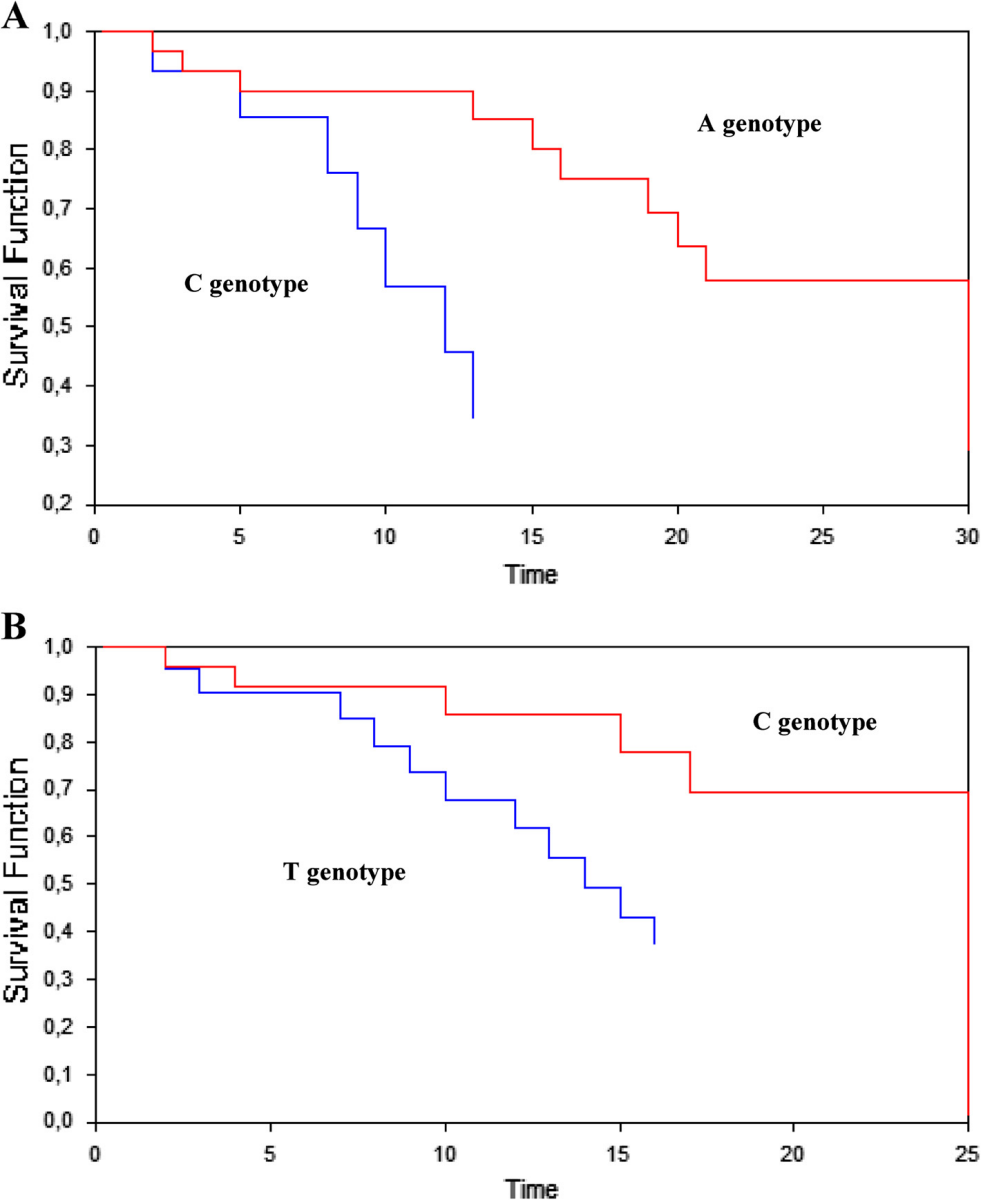
**Kaplan-Meier curves for ERCC1 C8092A (A) and T19007C (B) polymorphisms in T4 breast cancer patients.**

Using the Kaplan-Meier method, survival curves indicated that patients carrying either 8092A or 19007C genotypes presented a statistically-significant better overall survival in comparison with those carrying the remaining other genotypes (p < 0.001 for both 8092A and 19007C genotypes; Figure 1).

Using the Cox model adjusted according to age at diagnosis for a multivariate analysis, pathological response to primary chemotherapy and both two *ERCC1* polymorphisms remained the only parameters with a significant impact on prognosis in our series of breast cancer patients; no other association with overall survival was observed for the remaining variables (Table 3A). Again, the combined 8092A/19007C genotype remained a statistically independent factor predicting a more favourable prognosis in multivariate logistic regression (p = 0.049) (Table 3B).

**Table 3:** **Cox proportional hazard model for multivariate analysis of different parameters**

**A**				
***Characteristics***	***p-value***	***Odds ratio***	***95% CI lower***	***95% CI upper***
Menopausal status	0.176	0.854	0.177	1.465
Age at diagnosis	0.283	0.916	0.110	1.906
ER	0.454	0.809	0.191	2.233
HER-2	0.483	0.971	0.117	2.602
Triple negative status	0.128	1.006	0.213	2.834
Tumor grading	0.982	0.968	0.194	4.075
Lymph node (N+)	0.644	0.873	0.125	3.614
**Pathological response to primary chemotherapy**	**0.043**	**5.274**	**1.388**	**26.173**
**8092A**	**0.048**	**2.690**	**1.091**	**9.053**
**19007C**	**0.031**	**4.177**	**1.226**	**23.131**
**B**				
***Characteristics***	***p-value***	***Odds ratio***	***95% CI lower***	***95% CI upper***
C8092 + T19007	0.430	1.266	0.334	3.231
**8092A + 19007C**	**0.049**	**4.377**	**1.031**	**23.112**
ER	0.332	0.793	0.143	2.655
PR	0.459	1.127	0.178	4.117
HER-2	0.139	0.699	0.166	1.223
Ki67	0.073	0.222	0.053	1.081
Pathological response to primary chemotherapy	0.051	0.565	0.136	1.377

Single (A) or combined (B) ERCC1 genotypes have been considered for statistical analysis. Significant associations are in bold. CI, confidence interval.

Finally, it is worthy to notice that the two patients who carried a *BRCA1* or *BRCA2* germline mutation presented the highest overall survival rates of the series (238 and 195 months, respectively).

## Discussion

In this study, two single nucleotide polymorphisms, C8092A and T19007C, in *ERCC1* gene were retrospectively evaluated for their association with the clinical behavior in a group of T4 breast carcinoma patients of Sardinian origin, receiving platinum-based chemotherapy. We have demonstrated that two genotypes within such polymorphisms have a role as independent prognostic factors for a more favorable clinical outcome in this subset of patients.

In breast cancer, the choice of cytotoxic chemotherapy is generally based on tumor extent and disease features. Identification of surrogate markers as potential predictive factors will be useful to improve the clinical management of breast cancer patients (i.e., to identify which subset of patients is expected to show either a response or a lack of response to a particular therapy). Various attempts have been made to improve the survival of patients with T4 breast carcinoma, for instance, discovering novel predictive biomarkers to identify patients who may really benefit from platinum-based chemotherapy.

Platinum agents such as cisplatin and carboplatin are DNA-damaging agents with activity in breast cancer, particularly in the triple negative subgroup [34-36]. In neoadjuvant strategies for the treatment of locally advanced breast cancer (LABC), the utility of platinum agents in addition to standard chemotherapy is yet to be completely clarified. Therefore, identification of factors relevant to better predict the clinical outcome in response to platinum agents may be helpful in achieving an absolute advantage for the management of the LABC disease.

ERCC1 (excision repair cross complementation group 1) can recognize and correct DNA damage through a DNA strand incision and homologous recombination mechanisms [8,30]. High ERCC1 expression has been demonstrated to be associated with resistance to platinum-based chemotherapy and worse prognosis in cancer patients [20,37,38]. To this aim, we here assessed that the *ERCC1* gene polymorphisms may play an analogous clinical role as predictive and prognostic factor among patients with T4 breast cancer receiving platinum-based therapy.

Several clinical studies have explored the role of ERCC1 as a marker for platinum sensitivity in cancer patients. For example, various studies have focused on the relationship between ERCC1 polymorphisms and prognosis for the treatment with platinum agents in colorectal cancer patients. Sequence variations in *ERCC1* gene may indeed alter the DNA repair capacity, making biologically plausible to assume that polymorphisms of this gene might have functional significance in cancer.

In our study, the *ERCC1* polymorphisms were determined using an automated sequencing method. For C8092A polymorphism, the A genotype was significantly associated with overall survival of T4 breast cancer patients treated with chemotherapy containing platinum compounds (OR 1.957, 95%CI 1.276- 5.675; p value = 0.036). Analogously, the C genotype of T19007C polymorphism was significantly associated with overall survival in the same series (OR 3.875, 95%CI 1.865-18.851; p-value = 0.004). Univariate and multivariate Cox regression analyses showed that the combination of the 8092A and 19007C genotypes acts as an independent prognostic factor in this group of T4 breast cancer patients receiving platinum-based chemotherapy (p-values = 0.022 and 0.049, respectively).

The precise mechanism by which the C8092A polymorphism is positively associated with a favorable prognosis remains indeterminate, as there are no direct functional data available for this polymorphism. Since the SNP is located at the 3′untranslated region (3′-UTR) which can be controlled by regulatory proteins and micro-RNAs, the C8092A polymorphism in the 3′-UTR region could however affect the stability and, thus, translation rates of the corresponding mRNA, finally influencing the expression levels of the ERCC1 protein into the cells. On the other side, the synonymous T19007C polymorphism at codon 118 (Asn118Asn) is a common silent substitution, and the exact function of Asn118Asn has not been clarified yet. Again, it could also affect the stability of mRNA or influence the rates of translation by converting a high usage codon to a low usage one. Alternatively, it could be biologically plausible that this correlation may be mediated by linkage disequilibrium with other potentially functional single-nucleotide polymorphisms.

The entire group of 47 patients was then screened for germline mutations in *BRCA1/2* genes and, of note, two (4.3%) carriers of *BRCA1/2* mutations presented the highest rates of overall survival within the series. No statistical analysis was carried out due to such a limited number of *BRCA*-mutated cases. The lack of functional BRCA (mainly, BRCA1) can lead to increased sensitivity of the tumor cells to molecular damage, demonstrating that BRCA mutations represent a predictive marker of response to DNA-damaging chemotherapies [39-41]. Certainly, the reduced ability to repair damaged DNA along with the impairment of the function of the ERCC1 protein, due to the presence of the combined 8092A and 19007C genotypes, may have contributed to determine the longest overall survival in such a limited subset of patients from our series.

Several limitations of this study should be addressed. Firstly, the sample size may limit the statistical power of our study (findings need further replication in a larger patients’ collection); next, the belonging of cases to a particular, genetically-homogeneous population (ethnicity may interfere with observed associations in multifactorial diseases; extension of the study in other geographical areas with general, genetically-heterogeneous populations is also recommended). However, to our knowledge, this is the first study clearly demonstrating that polymorphisms in *ERCC1* gene are significantly associated with overall survival in patients with T4 breast cancer receiving platinum-based treatment. Overall, this seems to suggest that such sequence variations could be considered as novel prognostic biomarkers for the management of the T4 breast cancer patients.

## Abbreviations

DHPLC: Denaturing high-performance liquid chromatography
ER: Estrogen receptor
ERCC1: Excision repair cross-complementing 1
HER-2: Human epidermal growth factor receptor-2
LABC: Locally advanced breast cancer
NER: Nucleotide excision repair
OS: Overall survival
PR: Progesterone receptor
SNP: Single nucleotide polymorphism
